# Rhino-orbito-cerebral mucormycosis

**DOI:** 10.1016/S1808-8694(15)30505-X

**Published:** 2015-10-19

**Authors:** Igor Teixeira Raymundo, Beatriz Gonzalez De Araújo, Carina De Carvalho Costa, Joana Pinho Tavares, Cleyverton Garcia Lima, Luiz Augusto Nascimento, C. Galindo

**Affiliations:** 1 ENT resident physician - Brasilia University Hospital; 2 Otorhinolaryngologist - Brasilia University Hospital; 3 Otorhinolaryngologist - Brasilia University Hospital; 4 ENT resident physician - Brasilia University Hospital; 5 Radiologist - Brasilia University Hospital; 6 Head and Neck Surgery - Brasilia University Hospital. Hospital Universitàrio de Brasìlia - Universidade Federal de Brasìlia

**Keywords:** amphotericin b, child, diabetes mellitus, endoscopy, mucormycosis

## INTRODUCTION

Mucormycosis is the most lethal fungal infection in humans and it is manifested as a quick, progressive and invasive sinusitis. It usually affects immunocompromized patients. Mortality is high, despite early diagnosis.^1^, ^2^

We hereby present a severe case of a child with rhino-orbital-cerebral mucormycosis; however with a very favorable outcome after aggressive treatment.

## CASE PRESENTATION

A 12-year-old female patient started with sudden abdominal pain, polyuria, polydipsia, anorexia and a drop in conscience level. She was diagnosed with untreated type I diabetes mellitus (diabetic acidosis), with blood sugar of 508 mg/dl upon hospital admission. After one week in the hospital, she started having eyelid ptosis, facial pain, converging strabismus, left side epistaxis and a progressive worsening of her general health.

**Figure 1:**
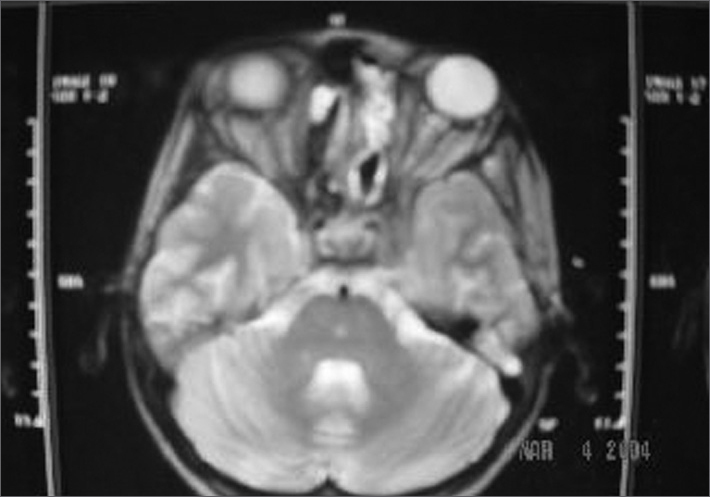
Soft tissue tumor occupying the left nasal cavity and invading the cavernous sinus.

Skull and paranasal sinuses CT scan and MRI showed a soft tissue mass in the left nasal cavity, invading the ethmoidal, maxillary and cavernous sinuses and the ipsilateral orbit (Figure 1).

An endoscopic-guided tumor biopsy showed mucormycosis. The patient was started in high doses of endovenous amphothericin B liposome for 45 days. There was a mild improvement only. Anterior rhinoscopy showed a large brown-greenish mass occupying the entire left nasal cavity.

We decided to do a nasosinusal endoscopic exploration. During the procedure we noticed a large mass coming from the maxillary sinus and the left nasal cavity. We did an exhaustive surgical debridement and cleaning in the maxillary, ethmoidal and sphenoidal sinuses with hypertonic saline solution, and we also flushed the region with an amphothericin B solution. Histology revealed a zygomycosis by Mucor genus fungi.

The patient had a favorable outcome and returned in one week for a review. Upon anterior rhinoscopy, we noticed a fungal mass. A new CT scan suggested tumor recurrence. The patient was then submitted to 29 daily sessions of oxygen therapy in a hyperbaric chamber and the amphothericin B was exchanged for intravenous itraconazole because of the long term use of the former, and she was also submitted to a rigorous diabetic control.

There was a mild improvement and stability on her condition, leading us to choose to do a second look endoscopic exploration. The left nasal cavity was broad and clear, without any evidence of infection. The maxillary sinus ostium was open and there were no signs of mucormycosis.

The patient was discharged from the hospital in a stable condition and using oral itraconazole. The endoscopic view confirmed disease clearance.

She is currently asymptomatic. Her capillary glucose is strictly controlled. She has nerve palsy of the III and IV cranial nerves on the left, however with good visual acuity and no diplopia.

## DISCUSSION

Mucormycosis or zygomycosis is a rare opportunistic disease caused by Mucorales fungi, which belong to the geni Mucor, Rhizopus, Rhizomucor, Absidia and Apophysomyces. The most virulent and frequent species is Rhizopus oryzae, which invades and clogs blood vessels, causing tissue schemia.^1^, ^2^ This disease can manifest itself in different clinical signs and symptoms, and the rhino-orbit-cerebral zygomycosis is the most severe form, manifesting itself usually in cases of diabetes mellitus of difficult control.^3^

Transmission is presumably through the inhalation pathway.

Many clinical conditions are associated with mucormycosis, but there are only two factors clearly associated with predisposing patients to the disease. The first is metabolic acidosis, and the second is disorders of neutrophils and monocytes.^4^

As a general rule, mucormycosis treatment is multimodal and includes the use of systemic antifungal drugs, surgical debridement and clinical control of the condition which is predisposing the patient to the disease.^3^

## FINAL REMARKS

Proper Rhino-Orbit-Cerebral Mucormycosis control includes early diagnosis, baseline disease control, treatment with antifungal systemic agents, proper sinus and orbital drainage and excision of the necrotic tissue, besides hyperbaric oxygen therapy.^5^
